# The Legislative Control of Medical Practice

**Published:** 1894-08

**Authors:** 


### *EDITORIAL.*

---

The office of THE JOURNAL is in room 330 Equitable Building.

Address all communications, and make all remittances payable to THE ATLANTA MEDICAL AND SURGICAL JOURNAL, Atlanta, Ga.

Articles for publication should reach this office not later than the 15th instant.

The editors are not responsible for views expressed by contributors.

---

### THE LEGISLATIVE CONTROL OF MEDICAL PRACTICE.

The above is the title of an excellent address lately delivered by Dr. Reginald H. Fitz, of Boston, before the Massachusetts Medical Society*.

This is an important matter to us as physicians, to those who wish to see the practice of medicine conducted only on a legitimate and honest basis. We in Georgia are particularly interested in it. We have already had much to say on this subject in these pages, but offer no apology for the following analysis of Dr. Fitz's admirable address.

The demand for legislative control of medical practice is apparent from the fact that "Patients are at times treated with reckless ignorance or negligence, and die in consequence, and no verdict of homicide is rendered; that ignorant and unskillful pretenders do not know the symptoms of contagious disease, do not suspect its presence, and make no report to the proper authorities; that in some States any one who chooses may practice medicine; that any one may forge a title to which he has no claim; that he may advertise himself a graduate of any institution he prefers; that he may claim to have accomplished any number of cures of what have been

---

*Published in Boston Medical and Surgical Journal, June 14 to July 12.

pronounced incurable disease; that he may promise specifics against any and all maladies; that he may publicly announce the most glaring untruths, all for the sake of deceiving and fleecing a credulous public, and the law cannot interfere with him." The law makes no distinction between regular physicians, homeopaths, eclectics, faith-curers, mind-healers, christian scientists, etc. Under the law all are alike physicians. But since so many stupid, ignorant and dishonest pretenders to practice exist, "it is clearly the duty of the State to discriminate, to legally qualify those who deserve the confidence of the people, and to disqualify those who are often the abettors of crime, the victimizers of youth, and the constant source of danger to every member of the community." The object of such legislation is to protect citizens by giving them a means of deciding to whom they shall apply for intelligent, skillful and honorable aid in time of need.

The opponents of medical legislation have been made up of those who practice some peculiar ism in medicine, the representatives of low-class medical colleges, the great army of medical black-legs, bunco-doctors, charmers, etc., with their hired attorneys. This lovely combination has tried to argue that "medical legislation invades personal liberty; is class legislation; tends to obstruct the progress of therapeutics; is unnecessary and not wanted; and has proven a failure." Dr. Fitz disposes of all these wild assertions with arguments that cannot be controverted.

Dr. Fitz then goes over the history of medical legislation in this country from the earliest legislation in the colonies (in Virginia) in 1639 down to the present time. In the first forty years of the present century attempts to regulate the practice of medicine were frequent and various. The opposition then, as now, came from quacks and pretenders. (In Georgia the legislature of 1825 made a law to "regulate the licensing of physicians." It was repealed in 1836 through the influence of the Thomsonians, steam-doctors, etc.) The author discusses also the rise and progress of Thomsonianism, with its relation to the efforts being made to secure proper legislation in the States. The ignorant followers of this new ism opposed all attempts at legislation, so that in 1850 there were practically no efficient laws controlling the practice of medicine. Since that date laws more or less efficient and satisfactory have been passed in nearly all the States. In some States there is no law yet; in others, the possession of a diploma and its registration suffice. In others the diploma must be examined and verified by a board. In others personal examinations are the sole qualification for license.

The laws in Alabama, Minnesota and Virginia are almost perfect. Those in North Carolina, Illinois, Iowa, Kentucky, Missouri, West Virginia and Indiana are meeting with general favor.

What, now, are the essentials of a law regulating the practice of medicine? The law must be executed by trustworthy citizens, properly qualified, and necessarily physicians. The experience of the majority of the States is in favor of the appointment of the controlling board by the governor of the State. They should be intelligent, educated, fair-minded, honest and upright representatives of the profession. They should be practitioners and not teachers, and should have been in practice several years. They should be chosen from different sections of the State. The Board of Examiners should be independent of the Board of Health. A single board, with proportionate representation of regulars, homeopaths and eclectics, is better than three separate boards. With the latter there will probably be separate standards of proficiency. A single board has the advantages of simplicity, uniformity of standard, and freedom from partiality.

After many years of labor and waiting, they have just secured a medical law in Massachusetts providing for the "registration of physicians and surgeons." It empowers the governor to appoint a Board of Registration, to be composed of seven persons, all of whom shall have been actively engaged in the practice of medicine for ten years. Persons now practicing medicine, graduates of a legally chartered medical college or university having the power to confer degrees in medicine, and every person who has been a practitioner of medicine in the State for a period of three years, next prior to the passage of this act, shall be entitled to registration without examination. Any person not entitled to registration under the above provisions must be examined. If found qualified he receives a certificate, and is duly registered. If he fails he may apply within two years for a second examination.

Examinations shall be, in whole or in part, in writing, and shall be of an elementary and practical character. They shall embrace the general subject of surgery, physiology, pathology, obstetrics and practice of medicine, and shall be sufficiently strict to test the qualifications of the candidate as a practitioner of medicine.

Violators of the provisions of this act shall be punished by a fine of not less than $100 nor more than $500 for each offense, or by imprisonment in jail three months, or both.

While the above is certainly a step toward proper medical legislation, we fear it does not go very far. One of the sections of the act affords too much latitude to "clairvoyants, and persons practicing hypnotism, magnetic healing, mind cure, cosmopathy," etc. Through these exemptions many frauds will continue their mean business in Massachusetts, and the object of the law will be to this extent defeated.

